# Supplementary material for: Cytomulate: accurate and efficient simulation of CyTOF data
Source: Genome Biol. 2023 Nov 16;24:262. doi: 10.1186/s13059-023-03099-1 (PMC10652542; doi:10.1186/s13059-023-03099-1)
Supplement: Supplementary file 1 — Additional file 1: Table S1. A table that provides the dataset name, species, the anatomic site, and its source for accession. [file 13059_2023_3099_MOESM1_ESM.pdf]

Dataset	Species	Anatomic Site	Source	Number of Markers	Number of Cells Events	Number of Cell Types
Levine_32dim	Human	Bone Marrow	HDCytoData	32	72,463	32
Levine_13dim	Human	Bone Marrow	HDCytoData	13	167,044	24
Samusik	Mouse	Bone Marrow	HDCytoData	39	53,173	24
CyAnno	Human	Peripheral Blood	https://flowrepository.org/id/FR-FCM-Z2V9	39	123,033	39
Covid	Human	Peripheral Blood	https://dbai.biohpc.swmed.edu/cytof-database.php	43	208,712	21
LG	Mouse	Lacrimal Gland	http://flowrepository.org/id/FR-FCM-ZY4P	31	284,055	7

Table S1. A table that provides the dataset name, species, the anatomic site, and its source for accession.
