# Supplementary material for: Cytomulate: accurate and efficient simulation of CyTOF data
Source: Genome Biol. 2023 Nov 16;24:262. doi: 10.1186/s13059-023-03099-1 (PMC10652542; doi:10.1186/s13059-023-03099-1)
Supplement: Supplementary file 3 — Additional file 3. Peer review history. [file 13059_2023_3099_MOESM3_ESM.docx]

**Review history**

**First round of review**

**Reviewer 1**

The paper proposes Cytomulate, a simulator that generates CytoTOF data using either based on user-defined settings or learned from a real dataset. The former Creation Mode enables users to simulate data de novo, while the latter Emulation Mode learns from an input expression matrix. The simulator uses a Gaussian mixture model, which the authors claim models well on transformed CytoTOF data, but we have doubts on this assumption (see point 3 below).
The authors used comparison to show that scRNA-seq simulators are unsuitable for CytoTOF data, but the conclusion is only based on Splatter (see point 5 below). Although the simulator has useful features (e.g., temporal effects due to instrument drift, batch effects, and cell differentiation) and the writing is clear, we have the following concerns.

Major:

1. We are not sure about the broad impacts of this simulator. The computational methods designed for CyTOF data are much fewer than those for other single-cell genomics. Compared to other single-cell omics (e.g., scRNA-seq), the feature dimension of CyTOF is much lower (only tens of proteins, thus a simpler analytical task), which restricts the needs of many computational methods (e.g., feature selection, and dimensionality reduction). The Cytomulate starts with preprocessed data, making it unsuitable for benchmarking different preprocessing procedures. It would be important for the authors to show more applications where Cytomulate can help benchmark CyTOF computational tools.

2. One suggestion is to assess how well the simulated expressions are clustered based on the cell type or subtype ground truths upon visualization via UMAP.

3. The normality assumption lacks justification. The roughly elliptic shapes in PC space are not strong evidence. We suggest the authors perform normality checks or at least visualize the marginal distributions.

4. The robustness test of Cytomulate on input cell type labels is not persuasive. The authors only included one special case for justification. It would be clearer if the authors include more numerical examples, like by randomly masking some cell type labels, and compare Cytomulate's performance using the randomly masked cell type input with that using the full cell type annotation.

5. If the authors want to compare Cytomulutate with other scRNA-seq simulators, they may choose those with better performance in benchmark studies (e.g., ZINB-WaVE, scDesign2, etc.). In benchmark studies, Splatter does not perform well in resembling real data; see Yue Cao, Pengyi Yang, and Jean Yee Hwa Yang. A benchmark study of simulation methods for single-cell RNA sequencing data. Nature communications, 12(1):1-12, 2021.; Helena L Crowell, Sarah X Morillo Leonardo, Charlotte Soneson, and Mark D Robinson. Built on sand: the shaky foundations of simulating single-cell RNA sequencing data. bioRxiv, 2021.)

6. While the authors used 6 datasets to show that their simulator can generate realistic data without cell identification, there is no evaluation of whether their simulator can mimic real data when cell types are used as input. We think it is important to incorporate cell types as ground truths for simulated data generation because many benchmark studies rely on the cell type ground truths.

Minor:
1. The usage of ranks in Fig 4e is unclear. Would using "scores" be a better choice?

2. It is not clear how the cell types are simulated in Fig 5a-e, please differentiate the cell types.

3. Quantitative measures should be used instead of qualitatively describing the clusters in Fig 5a-e.

**Reviewer 2**

This study is presenting a novel approach which allows the exploration of generation of simulated CyTOF datasets that capture characteristics of CyTOF data. Such simulations tools have been already developed for scRNA-seq data but not for CyTOF. Their method will allow the generation of several CyTOF datasets as a foundation for developing and validating several CyTOF data analysis tools. This aim is considerable in the field of CyTOF studies and the method they have developed seems to be appropriate for such aim.

The software is easy to install and use. However from the webpage and the material provided, there are some details missing to allow replication and comparison of the related analysis. It will be useful to provide the scripts used to reproduce the whole analysis (including the other methods) on the webpage and especially to be able to reproduce all the metrics computed.

Key remarks:

* Characteristics of CyTOF data
Some changes would improve this paragraph. It would be best to focus on describing characteristics of CyTOF data instead of assessing whether current scRNA-seq models would apply.

In the section "High volume, high dimension, and heterogeneity" you mention "heterogeneity" in the title, but you don't show or refer to any heterogeneity in the paragraph. Also, the last sentence L. 109-111 does not relate to previous, I don't understand the "therefore".

Similarly, you are explaining that the zero-inflation model does not apply to CyTOF data as it does in scRNA-seq. How about focusing on the characteristic of CyTOF data such as the fact that some proteins are too scarse to be detected and give more details about that.

The section "Dependent features" should be changed to the correlation in the protein-protein expression. Alternative if you do want to look at dependent features, you could have incorporated all/partial possible feature-feature correlations, or feature-feature-feature triple associations etc...

The section "Temporal effect" and "Cellular trajectory" should be shown from real data not from the simulations.

* Cytomulate Framework
This paragraph would benefit some more explanations and clear definition of the objective of each mode of simulation. From my understanding, there are two objectives:
- to produce one or more synthetic datasets that looks like a particular CyTOF dataset
- to capture certain characteristic/features of a dataset but not others
I would suggest adding the concise and clear objectives of each mode in L. 165-166.

The figure 1A needs some improvement to be clearer. It is difficult to understand what's the difference between Emulation Mode and Creation mode and needs to be clarified.
I understand that on the left inside is the input data and on the right inside the matrix is the output data, could you add some text to explain what is what?

L. 170: Could you please refer to the exact same characteristic names that were listed in the previous section, "column dependencies" was not mentioned before. I would suggest to list here all the characteristic/features that were described earlier and if some of the them were not taken into account, explain why.

L. 208-210: This has not been carefully explained/stated in the previous section.

L.166 and L.211: It would be useful to define "granular subtypes".

L.218-221: I would move this section before description of each mode to explain what is required for the simulation.

* Simulation Comparison Study

In the Figure 4 and Figure 5 the results obtained with Cytomulate do not reproduce the two-mode on the density map that are found on the real data. Why is that?

L.246-249 and L. 276: It would be useful to make the link with what has been described so far with protein channels correlations and the diagonal covariance.

The metrics that were selected have been computed at cluster level from what I understand from the method section and it should be specified in L. 291. Did you also look at the distribution at the single cell level?


Minor revision:
L.68 "However the current lack.." missing verb
L.81-83 The orthograph and structure of the sentence needs to be reviewed
L.109 "CyTOF" you should add experiment or dataset?
L.162 what do you mean by "class-leading efficiency" and where is it explained?
L. 163 I think "for any research need" is a strong statement.
L. 725: I found this ranking is not so intuitive, the fact that 1 rank is worse than rank 5.

**Reviewer 3**

**Review of “Cytomulate: Accurate and Efficient Simulation of CyTOF data”**

General comments:

1. The method proposed is almost the only method to simulate CyTOF data. It would be greatly useful for the study related.
2. It would be beneficial to provide an example that demonstrates how the proposed method improves upon existing analysis results in real data.
3. The enumeration method is understood as a model-based bootstrap procedure with several (could be strong) assumptions. I do wonder what are the advantages of the proposed emulation over the single or multi-split of the data with no assumptions?

Detailed comments:

1. Line 76, page 4: The authors say the CyTOF is more closely related with phenotype data. Does Cytomulate also simulate the relationship between two?
2. Line 79, page 4: The Cytomulate simulates the transformed data instead of the original count data, which are real valued due to the observational noise and normalization steps. I do agree the simulation of dependent multivariate count data is not easy. However, there are some good ones based on multivariate normal random variables. One I remember is “Simulating dependent discrete data” (2013, Madsen and Birkes, Journal of Statistical Computation and Simulation 83 (4):677–91), which uses the Gaussian copula.
3. Line 104, page 7 and Line 571, page 27: This is about normality or approximate nor- mality of the data. I do not think the PCA plot is enough to show the near-Gaussianity of the data. The PCA plots the weighted sum of variables and it is close to the normal even the data are count or non-normal. Any other supports?
4. Line 212-213, page 11: This is just a comment. I think the use of GMM (in emulation mode) still requires to know the mixture variables, the mixture probability and the number of mixtures. They would not be easy to estimate from the data when the dimension is large or high. I find the authors discuss this issue in Conclusion section.
5. Line 290, page 14: “Cytomulate well approximates real data”. It is not easy to un- derstand the similarity of two high dimensional data sets. I recommend the use of a metric based propensity score that is popular in synthetic data literature. The de- tails of the method can be found from Section 3 of “Assessing, Visualizing and Im- proving the Utility of Synthetic Data” by Raab, G.M., Nowok, B., and Dibben, C. <https://arxiv.org/abs/2109.12717>.
6. Lines 501-507, page 24: “A crucial advantage of employing *· · ·* but also produces sen- sible results for practitioners.” Any support or justification for this claim?
7. Line 637, page 31: “the L2 distances among observed average protein expressions of all cell types are used as weights.” I do wonder why the authors choose this type of edge weights.
8. Line 650, page 31: I do expect a few more lines on the generation of the cell trajectories using Brownian Bridges.

**Authors’ response to reviewers**

**Reviewer #1**: The manuscript Acute expression of human APOBEC3B in mice results in RNA editing and lethality demonstrates for the first time experimentally that overexpression of human A3B is lethal in an animal model. Further, this is the first study to show that A3B functions as an RNA deaminase. Overall, the data are convincing, interesting, and novel. While the study is largely descriptive/phenotypic, it provides a foundation for further research in the field to determine the mechanism of animal death from A3B overexpression and also the effect of RNA editing on the animal. The data spur interesting biochemical questions about the RNA substrate preference of A3B. This is a well-written and timely manuscript which has my support for publication with a few minor suggestions listed below.

RNA editing is not intuitively associated with driving cancer or other diseases. It would be nice to have a hypothesis for this mechanism stated in the introduction, to set the stage for subsequent data and discussion.

We thank the reviewer for the suggestion and have added the following sentence (line 57): “Even though most edits occur in non-coding regions and could have no impact, these modifications may trigger novel splice sites, alter microRNA activity, and/or mRNA stability. Interestingly, it has been shown that the impact of RNA editing on proteome diversity is comparable to, or potentially even greater than, that of somatic mutations (Gassner FJ et al., Peng et al.)”

Line 29-32: RNA editing is a dynamic process, but the RNA edits don't necessarily disappear right after the responsible enzyme is gone. Loss of RNA edits also requires turnover of transcripts. I suggest a clarification of this statement.

We have modified this sentence in the manuscript, (line 85): Unlike DNA mutations, RNA editing is a dynamic process which does not leave a permanent footprint and RNA edits disappear right after the responsible enzymes are no longer expressed or after the turnover of the edited transcripts.

Supp Fig 1E - the dots representing A3B levels on the far right are hard to see and colors are not discernable.

We have included a clearer version of this figure.

Fig 3B - please clarify in the legend and methods how relative contribution is defined. Is this the absolute amount of RNA edits in A3B mice? Or is it measured relative to control mice?

We are sorry for the confusion. RNA editing events included in this study were primarily extracted by removing the matched exome and transcriptomic data for each sample. Subsequently, the RNA editing per trinucleotide profiles for all base substitutions were plot as the absolute amount of RNA edits in A3B mice. Relative contribution refers to the contribution that each single base substitution has to the overall base substitution spectrum in A3B mice. We have clarified this in the figure legends and the methods.

Supp Fig 4 - the color coding is a little confusing because it encompasses both location and type of mutation but makes them mutually exclusive. These categories could co-occur (for example, a missense mutation in the 3'UTR) but the table does not allow for that information to be conveyed.

The reviewer is right. We intended to represent the annotation to better see the distribution of mutations. We have clarified this in the figure and correspondent figure legend, and changed “location” to “annotation”. Please note that Figure S4 is now Figure S5.

**Reviewer #2**: The manuscript by Alonso de la Vega and Colleagues reports the effects of APOBEC3B artificial induction in transgenic mice. The mice were generated by introducing an expression cassette bearing the coding sequence for an APOBEC3B-turboGFP chimera into the ColA1 locus under the control of a TetOn inducible promoter. The experiments were carried out after induction of the APOBEC3B chimera in 4-weeks old mice through doxycycline feeding.

The expression levels of the chimera were compared to the physiological/pathological ones in human tissues to make sure these were in the same order of magnitude. The two main findings are:

- prolonged expression of the APOBEC3B is lethal in mice

- with very high levels of APOBEC3B, its ability to target RNA becomes evident.

Overall, the manuscript is quite interesting: as it asks questions that have been lingering around for a while - specifically, how comparable are APOBEC3A and APOBEC3B, as they share a virtually identical C-terminal domain?  While a more exhaustive answer would have been preferable, the short answer is probably good enough: the two proteins are probably more similar than we would like to acknowledge, with the higher genotoxicity of APOBEC3B being the reason for these differences (APOBEC3B inability to trigger tumors in murine models and the failure to detect APOBEC3B dependent RNA editing in human cells).

Major comments

- As it might be relevant for DNA accessibility, it would be useful to show that localization of the APOBEC3B-turboGFP chimera is the same as that of wild-type APOBEC3B (and how it compares to APOBEC3A).

We thank the reviewer for this comment. We tried to generate cells that express wild-type APOBEC3B without turboGFP. Regrettably, when the plasmid was expressed in bacteria, A3B expression caused mutations in the bacterial DNA that prevented the bacteria to grow, making the amplification of the plasmid unachievable. Other reports have shown that A3B is expressed in the nucleus similar to what we also show in Figure S1C.

- How similar is the phenotype of APOBEC3B expressing mice to those from other genotoxic agents? If specific tissues/pathways that are responsible for the phenotype were identified for other genotoxic agents, it might be worth assessing them also in these mice.

This is an interesting idea. We suppose that, at some threshold, all genotoxic agents will become lethal to mice. We have not been able to address this idea here but parallel studies with animals constitutively expressing APOBEC3B (*Rosa26::CAG-A3Bi*) have demonstrated that this enzyme is capable of driving tumor development (Durfee *et al*., *bioRxiv* doi: <https://doi.org/10.1101/2022.04.26.489523>). Accelerated tumor development requires the enzyme’s catalytic glutamate (E255) and, interestingly, in preliminary studies that are ongoing, APOBEC3B and chemical mutagens appear to have synergistic effects. This work is being pursued by the Harris group at UT Health San Antonio and is expected to take at least another year to complete, and it is therefore well beyond the scope of the present study.

- Considering that RNA-seq experiments were performed using stranded libraries, there is no reason to group together C>U/G>A and A>G/U>C changes. This is quite important, as G>A and U>C changes can be used as internal controls for RNA editing. A useful way to depict the specificity of the RNA editing could be to plot each position with a change through its (average) frequency and its recurrence: only C>U and A>G changes should present a bias.

We thank the reviewer for pointing this out. We created a new R package called plotRNAedits to read mutation files and plot the full 192 context RNA edit profiles. The relevant figures have been included in Figures S3A and S3B for liver and pancreas samples, respectively. Plot RNAedits package is released through github and included in the methods section.

Minor points
- Is there any correlation between RNA editing levels and the expression levels of the edited transcripts – especially in the case of the recurrent sites?

We thank the reviewer for this comment. We have analysed the expression levels (TPMs) of the edited transcripts (for all the transcripts or only the recurrent ones) in liver, as well as in pancreas, and included these data in the revised Figure 4C. In the pancreas all the recurrent edited transcripts are more expressed than the ones that are not edited. Furthermore, all these editing events occur at the 3’UTR, which suggests that the editing might be affecting the stability of the edited transcript.

- It would be useful to add the editing frequency in Table 2 as well

We have now added the frequency in Table 2.

- It would be useful if the Authors could discuss how they explain the specificity/frequency of the editing at some of the sites with the requirement for a very high APOBEC3B expression. I would imagine that, with such specificity/frequency, even lower APOBEC3B expression would be sufficient to detect the editing.

This is another very interesting point made by the reviewer. In fact, editing does not occur when the expression of A3B is low. Apart from the one reported in this manuscript, we have generated an additional mouse model that expresses the rtTA transactivator under the control of the Rosa26 promoter: *TetO-A3B-tGFP/Rosa26-rtTA* (reported in Durfee *et al*., *bioRxiv* doi: <https://doi.org/10.1101/2022.04.26.489523>). After induction of doxycycline for 7 days, these mice expressed low levels of A3B in most of the tissues, in line with previous reports suggesting that the Rosa26 promoter has variable activity in different mouse tissues. Recurrent liver RNA editing events were validated in the livers of 2 mice after 7 days on doxycycline, in which no C-to-U changes were detected in any of the 7 positions examined, indicating that RNA editing activity requires high A3B expression levels. One hypothesis for this could be that A3B has more affinity for DNA substrates than RNA. Previous studies have demonstrated that A3A and APOBEC1 have a preferential targeting of DNA over RNA (<https://doi.org/10.1073/pnas.1306345110>, <https://doi.org/10.1093/nar/gkt945>, <https://doi.org/10.1021/acschembio.1c00919>). Therefore, low A3B levels could potentially focus on specific DNA substrates, whereas elevated A3B levels could deaminate a greater number of targets, including RNA molecules.

- page 6. "Feed ab libitum" should be "fed ad libitum"

We apologise for the mistake which we have now corrected and thank the reviewer for pointing this out.

**Second round of review**

**Reviewer 1**

In this version, the authors carefully addressed my previous questions. Based on their background introduction, CyTOF data is always pre-processed and is not at counts level anymore. scDesign2 shows reasonable results but is less optimal since it is a count simulator.

For the significance of the method, the authors admitted that the existing computational methods are much fewer than scRNA-seq, but they also added benchmarking on existing methods.

**Reviewer 2**

The efforts provided by the authors on this revision are really valuable. This revised version benefit from extra analyses which highlight the potential of the simulation tool. It is now also much clearer with more detailed explanations including some rephrasing of the text and the editing of the figures.

- Software and reproducibility: I appreciate that the authors provided the scripts in python and R used to reproduce the whole analysis (including the other methods) on their webpage.

- Characteristics of CyTOF data: I particularly like the details added on the preprocessing steps in a CyTOF system which are important to take into account. Most CyTOF users are not aware of those 6 steps and so far it has not been clearly stated in a publication.

- Comparing Batch Normalization Methods using Cytomulate:
The authors didn't mention CytofRUV (Trussart et al., Elife, 2020) and did not include it in their comparison. However, CytofRUV method has been shown to be outperforming CytoNorm (Trussart et al., Elife, 2020). It would be valuable to add this method with different k values in the benchmarking evaluation that has been provided.

**Reviewer 3**

The authors revised the manuscript appropriately and I do not have any other comments.

**Authors’ response to reviewers**

Reviewer 2: Comparing Batch Normalization Methods using Cytomulate: The authors didn't mention CytofRUV (Trussart et al., Elife, 2020) and did not include it in their comparison. However, CytofRUV method has been shown to be outperforming CytoNorm (Trussart et al., Elife, 2020). It would be valuable to add this method with different k values in the benchmarking evaluation that has been provided.

We thank the reviewer for pointing this out! In response to the reviewer’s comment, we have made several attempts in incorporating CytofRUV in our benchmark study on batch normalization. However, it was not trivial to construct .fcs files that were compatible with CATALYST which was used by CytofRUV. Respectfully, we did not include CytofRUV in our benchmark study due to the limited time frame. What’s more, while we agree with the reviewer that Cytomulate is a good choice for benchmarking existing methods in the field, it is beyond the scope of this paper to comprehensively benchmark methods for other workflows (normalization, etc). We hope that the results included in the paper will motivate other researchers to utilize Cytomulate for dedicated review/benchmark papers for data normalization in the CyTOF ecosystem.
